# Finding the right words: preparing medical students for communication on the wish for hastened death - medical students’ perceptions of drama-based and online communication training

**DOI:** 10.1186/s12909-026-10403-7

**Published:** 2026-09-19

**Authors:** M. Wegmann, M. Lemos, D. Fink, A. Scherg, F. Elsner

**Affiliations:** 1 Department of Palliative Medicine, Medical Faculty, RWTH Aachen University, Aachen, Germany; 2 Audiovisual Media Center, Medical Faculty, RWTH Aachen University, Aachen, Germany; 3 Palliativmedizin, Elbe Klinikum Stade, Stade, Germany

**Keywords:** Medical education, Assisted suicide, Hastened death, Communication training, Drama-based learning, Online learning, Hybrid teaching, Legal education, Palliative care

## Abstract

**Background:**

In Germany, assisted suicide is legal, placing ethical and communicative demands on physicians. Medical students (MS), however, often feel inadequately prepared for such conversations. This study explores how MS perceive two teaching formats designed to support communication skills in such a context.

**Methods:**

As part of the Erasmus+-funded ELPIS project (E-Learning on Palliative Care for International Students), in 2023 a drama seminar (DS) and an online tool (OT) were offered to MS of the RWTH Aachen University on a voluntary basis to support communication skills training. For evaluation, qualitative interviews were conducted with 15 MS from RWTH Aachen University. Semi-structured interviews were transcribed and analysed through qualitative content analysis, applying both inductive and deductive approaches.

**Results:**

All 15 MS viewed the DS as emotionally intense and immersive, with empathic engagement, self-reflection, and opportunities to practise communication skills emerging as central aspects of the experience. Several MS, however, reported emotional overload when performing in front of peers. The OT was appreciated for its flexibility, structured design, and emotionally safe environment, particularly by those with limited prior experience. Its limitations included reduced realism, lack of non-verbal interaction, and a diminished sense of authenticity. MS suggested a hybrid learning approach, using the OT as a preparatory phase before the DS. The interviews revealed a lack of knowledge regarding the legal framework of assisted suicide, reinforcing the need to integrate legal and ethical education into the medical curriculum.

**Conclusion:**

The findings suggest that the two formats offer different strengths in preparing MS for challenging end-of-life conversations, with the interview findings further indicating that a blended learning format may reduce emotional barriers and support a stepwise approach to communication training. Integrating communication training alongside ethical and legal education represents an important step towards strengthening palliative care curricula.

**Supplementary Information:**

The online version contains supplementary material available at https://doi.org/10.1186/s12909-026-10403-7.

## Background

In Germany, suicide has not been a criminal offence since 1871, and consequently assistance in suicide was and is also not punishable [1]. Assisted suicide refers to situations in which a person intentionally ends their life with the assistance of another individual, while performing the final life-ending act themselves [2]. People who are capable of making independent and responsible decisions also have the right to end their lives with assistance from a third party [3]. This decision led to a reform of the German Medical Association’s professional code of conduct in 2021, which entailed the removal of the previous prohibition of assisted suicide [4]. Since then, there has been an increase in requests for assisted suicide, which presents physicians with new ethical and practical challenges [5]. Physicians are required to discuss the patient’s wish for hastened death [4]. The aim of this is not just to share knowledge with patients, but also to take their wishes and fears into account [6, 7]. For many patients, the opportunity to talk about their desire to die is a significant source of relief [7]. Consequently, the acquisition of skills to communicate both empathically and respectfully in these situations should be a fundamental aspect of medical education. However, many MS do not feel adequately prepared for these challenging conversations. International studies have shown that palliative care and the communication of sensitive issues, such as patients’ wishes for hastened death, have increasingly been included in medical curricula. However, the extent and depth of training remain insufficient to meet the needs of clinical practice and the expectations of MS [8–11]. The discrepancy between the theoretical knowledge offered in medical education and the practical demands of later clinical practice remains significant. Limited time and resources for the development of communication skills in undergraduate education further exacerbate the issue, as education often places greater emphasis on theoretical content than on practical skills [12–14]. Various initiatives have been launched to improve training in palliative care communication skills. Since 2009, palliative medicine has been a compulsory subject in Germany [5, 15]. Responding to the increased demand and taking into account limited opportunities for real-life patient contact, online teaching methods have been developed in recent years. During the global COVID-19 pandemic, there was a shift towards the adoption of online tools (OT) [16, 17]. As a result, OTs are becoming an essential part of medical education. The ELPIS project, funded by the Erasmus+ programme, is an example of the efforts to improve in-depth training in communication skills in the context of palliative care for MS [14]. It explores innovative approaches to palliative care education, including OTs designed to support communication skills training. In summer 2023, as part of ELPIS, the study examined how MS experienced an in-person DS and an interactive OT designed to prepare them for difficult conversations about the wish for hastened death. This study was guided by one primary and three secondary research questions. The primary research question focused on how MS perceived the DS and the OT in preparing them for communication with patients about the wish for hastened death and assisted suicide. The secondary research questions addressed MSs’ perceptions of the current legal framework surrounding assisted suicide in Germany and related uncertainties. Further aspects included the ethical attitudes, conditions, and personal boundaries described by MS when dealing with wishes for hastened death and assisted suicide, as well as their evaluation of the current state of communication training in medical education regarding assisted suicide and other challenging end-of-life conversations.

## Methods

### Participants

The study sample comprised 15 MS in the 5th to 10th semesters of their medical studies at Rheinisch-Westfälische Technische Hochschule (RWTH), representing all students who voluntarily participated in the course and subsequently completed the interviews. The sample size therefore reflected the total number of MS participating in the course.

As participants were from different semesters, we expected varying levels of prior experience regarding conversations about assisted suicide. Some MS had already witnessed conversations about hastened death and assisted suicide through previous work as nurses, clinical placements, or private experiences. In addition, MS in their later semesters had completed in-person drama sessions as part of the mandatory palliative care teaching module. Given the exploratory qualitative design, the sample was considered sufficient to provide an in-depth insight into how MS at different stages of their medical education and with varying levels of prior experience viewed the teaching formats. Statistical representativeness was not an objective of the study. Throughout the study, the interviewer ensured that no personal relationship existed between herself and the MS to mitigate potential bias.

The study was reviewed by the Ethics Committee of the RWTH Aachen University (approved: EK 23–213, 14 July 2023). MS received information sheets, signed data protection agreements and provided consent for audio recording of the interviews and pseudonymisation. Each respondent was interviewed once.

### Setting

The course was offered during the summer term of 2023. The programme involved a two-hour DS and a two-hour OT, both followed by an online evaluation and concluding with a final interview. The online evaluation was analysed separately and is not part of the present qualitative analysis. Findings of the evaluation of the DS have been reported in a previous publication [8]. The interviews took place between November and December 2023, either in person or via a video-conferencing tool (Zoom). Interviews were scheduled using a web-based tool (Kulibri), were conducted in German, and lasted 30–45 min.

### Procedure

#### Educational Intervention

All MS completed the DS prior to the OT. Before both teaching formats, Dr. Scherg delivered an introductory lecture on the current framework surrounding assisted suicide in Germany. The lecture was presented live before the DS and provided as a pre-recorded video in the OT, in which MS were required to respond to questions to proceed with the presentation. During the DS, MS were able to ask questions in person.

For the DS, the MS were divided into two groups, with each group attending the seminar on a different day. Each role-play simulated a conversation between a physician and a lung cancer patient. One student assumed the role of the physician, who was also the patient’s godchild, while the remaining MS observed the interaction. The role of the godmother was portrayed by an external simulated patient. Each role-play lasted approximately 15–20 min and was followed by a feedback and discussion session, during which MS could ask questions, reflect on the conversation, and discuss communication strategies. The scenario was set during a birthday celebration. During the party, the godmother took her goddaughter, a physician, aside for a private conversation. She disclosed that she was in the terminal stage of lung cancer with no further curative treatment options and expressed a wish for hastened death, asking her godchild for support.

In the OT, which was developed by Martin Lemos and Daniel Fink, the same scenario was presented in an interactive digital format that MS could complete independently on a day of their choice. The module began with the introductory video establishing the birthday party setting, followed by the godmother expressing her wish for hastened death. The MS, taking the role of the physician godchild, then selected one of four possible response options. Depending on the selected response, the conversation developed differently. Unlike the DS, no feedback session was provided. However, the scenario could be repeated as often as desired, allowing MS to explore all available response options.

#### Interviews

Following completion of both teaching formats, semi-structured interviews were conducted. The interview guide, developed by the first author in collaboration with Prof. Elsner and based on the research questions, included open-ended questions on attitudes towards assisted suicide, prior experiences, the legal context, ethical boundaries, the physician’s role, preparedness through medical education, and perceptions of the teaching formats. The complete interview guide is provided in Supplementary Material 4.

All interviews were conducted, audio-recorded, and manually transcribed by the author, a medical student in her 8th semester at RWTH Aachen. Interview sequences were translated from German into English by the author. Field notes documented non-verbal communication, participants’ emotional responses, contextual observations, as well as the date, location, and duration of each interview.

### Data analysis

A qualitative content analysis was conducted using Johnny Saldaña’s two-cycle coding approach [18]. The software MAXQDA was used to support the coding process [19]. Throughout the analysis, memos were used to document reflections, emerging ideas, and methodological decisions.

The first coding cycle served as an initial orientation to the data. Preliminary analysis identified thematic areas before descriptive, in vivo, emotion, and values coding was applied to capture significant expressions and words used by the MS and to reflect their perspectives, emotional responses, and beliefs. In a subsequent step within the first cycle, these initial codes were grouped into broader categories. To provide a structured overview of the preliminary coding framework, the “MaxMaps Creative Coding Tool”, a digital mind mapping feature within MAXQDA, was used for visual representation.

In the second cycle, the initial codes were further refined through continuous review and reorganisation, enabling the identification of correlations between categories. These were organised into subcategories. As a final step, Saldaña’s “Operational Model Diagram” was used to visualise the relationships and interactions between the developed categories, providing an overview of the final coding framework. All coding was conducted by the first author, who also developed the initial coding framework. Dr. Scherg independently reviewed the coding, and selected coding decisions were discussed between both researchers. The coding framework was further refined by the first author in consultation with Dr. Scherg, while the final categories and subcategories were determined by the first author. The finalised coding system, including main codes and subcodes, is presented in (Supplementary Material 1: Supplementary figure 1. Final coding system and thematic structure).

## Results

The results are structured around the main themes addressed by the research questions (Supplementary Material 2: Table 1. Characteristics of participants). 

Each section is anchored by one representative quotation that best captures the respective outcome. Additional quotations supporting and elaborating on these themes are provided in (Supplementary Material 3: Table 2. Selected exemplary quotations).

### Drama seminar vs. online tool

This section addresses the primary research question by examining how MS perceived the DS and the OT in the context of difficult conversations about the wish for hastened death and assisted suicide, focusing on learning experiences with both teaching formats. The analysis comprises evaluations of the DS and OT, with a comparison of both.

#### Evaluation of the drama seminar

MS described the DS as both practice-oriented and emotionally engaging, with the realistic conversation scenario allowing them to practise communication skills within an immersive learning experience on both a cognitive and physical level.

*Field note (FN): “She appeared visibly uncomfortable*,* displaying an embarrassed smile and nervously shaking her legs.” (FN1)*

Observations revealed pronounced physical reactions, including tension, nervousness and discomfort, which were also reflected in MS’ own accounts.

*“I was really in the situation. I really had to get myself out of it afterwards. It was an absolutely realistic training situation.” (P10)*

In this setting, numerous MS reported gaining insight into their personal boundaries and ethical beliefs. This process of self-reflection was further supported by feedback from both peers and lecturers. The combination of professional expertise, characterised by its practical relevance, and the diverse perspectives of the MS was noted as conducive to reflection on their own communication approaches.

Despite the positive experience with the DS described by MS, not all found the active participation to be an entirely positive experience. For some MS, engaging in a sensitive conversation in front of the whole group was overwhelming. Conversely, other MS felt that they learned more through active participation than by observing from a distance.

Taken together, these experiences shaped MSs’ recommendations for future curricular development. A recurring theme across the interviews was the broad consensus that additional training opportunities of this kind are needed, with many MS describing a lack of preparation for mentally challenging conversations in the current curriculum. In this context, they proposed that such scenarios should be practised more often in small groups or one-to-one settings to enable more personalised learning and help prevent MS from feeling overwhelmed.

#### Evaluation of the online tool

In contrast to the DS, the OT was praised for its location and time flexibility. MS were able to incorporate a predetermined time frame into their daily routines, a factor which was acknowledged as both relieving and supportive. Completing the OT in a familiar environment was evaluated positively, as this setting provided a protected framework for engaging with the sensitive topic of assisted suicide.

In terms of structure, the OT guided the MS throughout the simulated conversation.

The predefined response options provided MS with a sense of orientation and security, particularly for those with limited prior experience.

Despite the flexibility of the OT, the absence of non-verbal interaction, such as facial expressions and body language, was criticised by all MS. As a result, the interaction was perceived as less realistic and more artificial.

*“Gestures and facial expression and body language*,* I don’t think you could get those at all. I felt much less involved (…).” (P10)*

Many MS found it challenging to establish an affective connection with the digital scenario and consequently considered the OT less memorable and emotionally intense.

Another common concern was the restricted opportunity for spontaneous expression. The inability to use individual phrasing was regarded as a limitation of authentic dialogue and was experienced as limiting the communicative depth of the scenario.

Based on their experiences with the OT, several MS proposed that the implementation of artificial intelligence (AI) could facilitate personalised dialogues. A video-based live conversation with drama patients was suggested, with the aim of creating a more authentic and emotionally engaging interaction.

#### Comparison of both forms of teaching

A clear preference for the DS emerged because MS experienced it as more authentic and emotionally engaging. MS frequently reported that the DS provided a more immersive dialogue experience, as the presence of a counterpart created a more engaging atmosphere.

*“But overall*,* I preferred the in-person version*,* because I think it’s easier to empathise with the other person when they’re sitting across from you.” (P11)*

MS identified direct engagement with affective reactions and non-verbal language as a central aspect of the learning process, particularly in relation to empathic understanding.

The DS also presented challenges. MS reported feeling under pressure in the unfamiliar dialogue situation. Several MS proposed using the OT as a preparatory measure to reduce fear of contact. MS also proposed that the introductory theoretical input should generally be offered online for both the DS and the OT, prior to the respective learning activities.

### Legal framework

Beyond the scope of teaching formats, the analysis delves into the way MS perceive the prevailing legal framework concerning assisted suicide in Germany.

#### The role of legal uncertainty

Uncertainty regarding the legal framework of assisted suicide emerged as a central theme in the interviews. Many MS indicated that there is a lack of clear knowledge about the current legal situation in Germany within the medical context.

Several MS reported that they had lacked reliable information about the legal status of assisted suicide and had been unaware of the current legal situation prior to the seminar.

*“I was totally surprised at the seminar. I didn’t realise*,* and neither did anyone around me*,* that assisted suicide is now legally permitted in Germany and that we are allowed to practice it.” (P10)*

#### Proposals for enhancing the assisted suicide act

To reduce uncertainty surrounding assisted suicide, MS voiced a desire for clearer legal guidance that also takes ethical aspects into account and provides orientation. Despite the need for flexibility, MS emphasised the need for a fundamental framework in a manner comparable to other medical procedures that demand guidance. Several MS questioned whether legal structures could address all cases fairly, describing a dilemma between individual patients’ needs and prevention of abuse through binding regulations.

*“You can’t really fit the dying process into some kind of fixed pattern. But somehow*,* there are certain points*,* certain standards*,* that help ensure that assisted dying isn’t prescribed or carried out without diligence.” (P5)*

In addition, MS called for legal regulations to protect particularly vulnerable groups, such as people with severe disabilities unable to initiate assisted suicide. These views suggest that some MS associated clearer legal guidance with protecting vulnerable groups while respecting patients’ self-determination. MS expressed support for assisted suicide as a means of preserving autonomy, while also emphasising the importance of legal certainty and guidance. Several MS associated clearer legal guidance with reduced uncertainty and responsibility when approaching these conversations.

### Ethical considerations in assisted suicide

This passage builds on legal considerations by exploring how ethical and practical considerations influenced MS’ sense of preparedness for conversations about wishes for hastened death in the context of assisted suicide.

#### Ethical self-determination and ethical values

The capacity of patients to make free and informed decisions was a significant priority for many MS when it came to recognising and accepting patients’ wishes for hastened death. Active involvement of patients in the management of their illness also stood out as a priority for MS.

*“(…) generally speaking*,* it’s about accepting that someone may have the wish to die*,* and that they might want to exercise control over the circumstances of their death. They don’t want to just leave it up to chance. (…) but rather want to die on their own terms.” (P12)*

MS indicated that an individual’s personal stance on assisted suicide might not always align with that of the patient. The challenge of balancing one’s own values and the wishes and needs of the patients concerned was articulated. A recurrent theme was the role of the patient’s age, with a few MS indicating that their reactions to the wish for assisted suicide might vary depending on the patient’s age, while some indicated explicit emotional boundaries in relation to patients’ age. MS expressed that advanced age is often associated with “end of life”, and that a wish for hastened death at a young age would be mentally much more challenging. An additional factor was the progression of the illness and the time that had passed since the diagnosis. Time in particular served as an indicator for some MS of the stability of the desire for assisted suicide rather than an expression of acute and potentially temporary despair. The ethical convictions held by MS were reflected in their understanding of health, dignity and suffering. One medical student expressed internal ambivalence: while confirming their commitment to the medical ideal of preserving life, they also viewed a wish for hastened death as an expression of autonomy, and a means of preserving dignity and relieving pain. Despite the diversity of views, MS commonly regarded a wish for hastened death as potentially legitimate, with their personal values and patients’ self-determination shaping their reflections.

#### Balancing responsibility and emotional demands

This section explores the psychological stress and responsibility involved in dealing with wishes for hastened death. Collegial support and teamwork in complex situations are emphasised.

*“I would find it difficult to deal with this in isolation as a general practitioner without perhaps having colleagues to support me and perhaps also pick me up if I can’t cope with it on my own.” (P11)*

The question of responsibility in the case of a wish for hastened death is addressed. A recurring issue concerned the extent to which the responsibility and decision-making should lie with the doctor or the individual concerned. Some MS reflected critically on situations in which responsibility might be shifted from the patient to the physician, and described this as ethically and emotionally challenging. The issue was considered especially significant when patients expressed a wish for hastened death and had a psychiatric diagnosis.

In this context, some MS indicated uncertainty regarding whether individuals with a psychiatric diagnosis could make fully autonomous decisions about their care. They raised concerns about denying access to assisted suicide when an individual’s illness affects their decision-making capacity. MS repeatedly emphasised the importance of self-protection. They identified the need to set personal boundaries, even if this might result in certain patient groups being excluded from assisted suicide, to cope with the emotional burden and responsibility inherent in such decisions.

#### (Free) decision-making

Many MS questioned whether a wish for assisted suicide could always be considered a freely responsible decision. External factors, such as family pressure and care needs, were mentioned as making the conversations challenging. The interview data suggest that assessing a “free” decision was regarded as a source of uncertainty when discussing wishes for hastened death.

*“(…) the reality is that resources are limited*,* and spots in nursing homes and hospitals are scarce. And of course*,* in an ideal world*,* you’d hope that wouldn’t affect the patient. (…) But we’re not free from pressure either. That too remains an idealised assumption.” (P5)*

The patient’s physical condition, including mobility and physical fitness, appeared to influence how MS assessed whether a wish for hastened death represented a free and responsible decision.

### Teaching on communication in the context of hastened death

The final section examines the experiences of MS in educational settings aimed at developing their communication skills. This examination is informed by a broader concern regarding the theoretical foundation of their education and its limitations in terms of practical application.

MS voiced critical views on the current approach to teaching challenging communication in existential situations. They argued that the curriculum places greater emphasis on theoretical content, whilst practical skills, such as interpersonal dialogue in difficult situations, are underrepresented.

*“(…) like in almost all areas*,* we receive much more theoretical than practical training. I mean*,* in general we have very little training in communication skills in anything like that. And we barely encounter the whole ethical or emotional side of things.” (P5)*

Differences in how MS approached patient interactions appeared to be associated with prior healthcare experience, as some MS reported having more practical exposure to patients than others. Some MS spoke about emotional challenges in medical work and voiced a desire for teaching that addressed strategies for coping with emotionally demanding situations. One medical student considered communication skills to be of less importance because MS spend little time with patients during their education and regarded communication as having minimal immediate academic benefit in a tightly structured curriculum. Nevertheless, the majority of MS expressed a strong interest in communication training, particularly with simulated patients.

MS expressed a desire for these initiatives to be expanded as part of their preparation for their future professional role, while also emphasising the importance of incorporating legal and ethical considerations, notably within the context of palliative care and assisted suicide.

## Discussion

Findings indicate that the two teaching formats possess different but complementary characteristics. While the DS was experienced as realistic and emotionally engaging, its intensity was also challenging for some MS. In contrast, the OT provided greater structure, flexibility, and a sense of security, leading several MS to suggest its use as preparation for subsequent in-person training. Beyond the teaching formats, legal and ethical uncertainties emerged as relevant aspects of preparing MS for conversations about wishes for hastened death and assisted suicide.

### Emotional engagement in communication training

The findings suggest that emotional engagement constitutes both a valuable and challenging aspect of communication training. The emotional intensity and sense of authenticity of the DS appeared to be closely interconnected, as direct interaction allowed MS to engage with both verbal and non-verbal aspects of communication. This is supported by the findings of a previously published evaluation of the DS, which indicated that “The majority of the students were emotionally moved by the conversations with the simulated patient.” [8]. However, it should be noted that this intensity can also become overwhelming for the students involved, particularly in situations requiring them to perform in front of their peers and faculty.

This dual role of emotional engagement is consistent with previous research indicating that emotionally charged learning experiences may support memory [20], while elevated emotional stress and anxiety can negatively affect students’ performance in simulated learning environments [21]. It is therefore reasonable to hypothesise that a certain degree of emotional tension may be an inherent component of realistic communication training. At the same time, it is important to note that excessive emotional demands have the potential to interfere with the learning experience. Beyond the emotional engagement, the opportunity for open group discussions and direct exchange with faculty was also valued by MS, reflecting previous findings on the potential of group discussions to enhance learning outcomes [22, 23].

### Complementary roles of digital and in-person training

In contrast, the OT, with its predefined response options, was less engaging and generated lower levels of emotional involvement in comparison with the DS. Some MS described the experience as “like a quiz”, indicating a sense of artificiality and emotional distance from the experience. The difficulty of replicating direct interaction and real-life circumstances has been identified as a recurring limitation in digital learning settings [17, 24]. This finding suggests that digital learning settings may be less suited to certain areas of medical training, such as training for emotionally complex conversations in end-of-life care.

It was posited by numerous MS that a hybrid approach could be beneficial, with the OT serving as preparation for the DS and helping MS to become familiar with emotionally challenging conversations. This approach aligns with blended learning methodologies, which integrate online learning with interactive in-person sessions [25]. The present study contributes to the international literature on medical education by offering qualitative insights into how MS perceive the respective strengths and limitations of digital and in-person training for emotionally and ethically complex conversations. In this context, the OT could offer a structured, safe introduction to sensitive topics and allow MS to consider different response options [23, 26].

### Online tools as a supplementary resource

In accordance with the international literature, MS identified flexibility in terms of time and location as a key advantage of the OT [17, 22], which may also support self-directed learning, allowing students to focus on specific areas according to their needs [22].

MS also emphasised the necessity for the medical curriculum to incorporate more communication training. Current research has indicated that consistent engagement in diverse conversation scenarios and case studies may support communication skills and preparedness for future clinical interactions [26]. However, the integration and expansion of practical communication training within medical curricula remain limited, with previous research pointing to restricted curricular representation and limited teaching resources [14, 27]. Digital teaching formats may therefore offer a supplementary learning resource that can be accessed flexibly and used with larger student cohorts, reducing reliance on additional teaching staff or simulated patients [22, 28–30].

A comparison of learning outcomes has been made between digital and conventional formats. However, the evidence available for the comparison is limited by methodological concerns and low study quality [17, 28, 31]. At the same time, OTs differ considerably in their objectives, content, and instructional design, thereby constraining the extent to which findings can be generalised across digital teaching formats [14]. The educational value of these tools is contingent on their design and implementation, as well as the manner in which they are utilised by students and instructors [26].

### Legal and ethical preparedness

Alongside communication skills, MS identified legal and ethical uncertainty as a challenge when preparing for conversations about wishes for hastened death and assisted suicide. The ethical concerns that emerged focused on questions of autonomy, responsibility, and free decision-making. Reflection on responsibility has been identified as an important component of communication training in medical education, particularly in the context of preparing MS for discussions concerning wishes for hastened death [32].

Uncertainty regarding the legal framework has also been observed beyond medical education. A 2024 Forsa survey found that only 15% of the general population were aware of the current legal status of assisted suicide in Germany [33]. Although not directly comparable, this suggests broader legal uncertainty and underlines the relevance of integrating legal and ethical aspects into communication training [5].

### Limitations

Voluntary participation may have introduced self-selection bias, as students with a particular interest in the topic or a more favourable attitude towards it may have been overrepresented, potentially influencing their level of engagement and recall. Moreover, MS were at different stages in their training, which may have influenced their familiarity with the subject matter.

External factors, including potential technical issues during OT use and the time gap between the interventions in summer 2023 and interviews in late 2023, may also have influenced MS’ perceptions and recall. While this interval may have affected the accuracy of recall, it also provided insight into which aspects of the training remained salient over time. In addition, MS completed the DS prior to the OT, which may have introduced a sequencing effect and influenced their perceptions of the subsequent format.

In qualitative research, data analysis inherently involves interpretative processes shaped by the researchers’ perspectives. To enhance rigour, systematic analytical procedures were applied, and the coding process was discussed with co-authors. The sample size and recruitment from a single institution further limit the transferability of the findings.

### Technological advancement as an opportunity

Recent technological advances have given rise to new opportunities in the field of digital communication training. A meta-analysis conducted nearly a decade ago may no longer fully reflect the capabilities of contemporary digital learning tools [34]. MS frequently identified the limited initiative and emotional responsiveness of the digital simulated patient as a limitation. AI-supported approaches may offer potential to enhance interactivity and responsiveness in digital learning environments [30]. This potential is currently being explored in an ongoing follow-up project by the authors. Previous literature has also emphasised the broader potential of AI-enhanced approaches in medical education [24, 30]. The potential of such approaches to address limitations identified in digital training for conversations about wishes for hastened death remains a subject for future research.

## Conclusion

The present study suggests that MS viewed both the DS and the OT as valuable resources for preparing individuals for conversations about wishes for hastened death and assisted suicide. However, it is important to note that each of these resources offered distinct strengths. The DS was commended for its emotional depth, perceived realism, and interactive learning opportunities, while the OT was acknowledged for its flexibility, structured format, and the sense of security it provided.

MS recommended that a combined approach be adopted, integrating digital tools with subsequent in-person training, for the purpose of preparing MS for challenging communication scenarios. The findings also point to gaps and uncertainties in MS’ understanding of the legal framework in Germany and emphasise the importance of ethical reflection, individual circumstances, and personal boundaries. MS expressed a need for enhanced communication training to be incorporated into medical education, in conjunction with the integration of ethical and legal content.

In view of the diversity of digital teaching tools and their varied instructional designs, further research is required to explore how different approaches can support communication training in specific educational contexts. AI-supported approaches may help address the limited interactivity and emotional responsiveness of current digital training formats, although their educational potential requires further investigation.

## Supplementary Information

Supplementary Material 1. Supplementary figure 1. Final coding system and thematic structure.

Supplementary Material 2. Table 1. Characteristics of participants.

Supplementary Material 3. Table 2. Selected exemplary quotations.

Supplementary Material 4. Semi-structured interview guide: *Finding the Right Words: Students’ Perspectives on Communication about Wishes for Hastened Death*.

## Data Availability

The interview transcripts and datasets generated and analysed during the current study are not publicly available due to the sensitive nature of the interview data and the need to protect participant confidentiality but are available from the corresponding author (MW) upon reasonable request.

## Abbreviations

DS: Drama seminar
OT: Online tool
FN: Field note
MS: Medical students
ELPIS: E-Learning on Palliative Care for International Students
P: Participant
