# Supplementary material for: Finding the right words: preparing medical students for communication on the wish for hastened death - medical students’ perceptions of drama-based and online communication training
Source: BMC Med Educ. 2026 Sep 19;26:1531. doi: 10.1186/s12909-026-10403-7 (PMC13613609; doi:10.1186/s12909-026-10403-7)
Supplement: Supplementary file 1 [file 12909_2026_10403_MOESM1_ESM.docx]

**Additional file 1:** Supplementary figure 1. Final coding system and thematic structure of the results
