# Supplementary material for: Finding the right words: preparing medical students for communication on the wish for hastened death - medical students’ perceptions of drama-based and online communication training
Source: BMC Med Educ. 2026 Sep 19;26:1531. doi: 10.1186/s12909-026-10403-7 (PMC13613609; doi:10.1186/s12909-026-10403-7)
Supplement: Supplementary file 2 [file 12909_2026_10403_MOESM2_ESM.docx]

**Additional file 2:** Table 1. Characteristics of participants

Table 1. Characteristics of participants

| Parameter |  | Number (n=15) |
| --- | --- | --- |
| Sex |  |  |
|  | Male | 3 (20%) |
|  | Female | 12 (80%) |
| Age |  |  |
|  | <20 | 1 (6.7%) |
|  | 20–25 | 10 (66.7%) |
|  | >25 | 4 (26.7%) |
| Semester |  |  |
|  | 5th semester | 8 (53.3%) |
|  | 7th semester | 5 (33.3%) |
|  | 9th semester | 1 (6.7%) |
|  | 10th semester | 1 (6.7%) |
| University |  |  |
|  | RWTH Aachen University | 15 (100%) |
