# Supplementary material for: Finding the right words: preparing medical students for communication on the wish for hastened death - medical students’ perceptions of drama-based and online communication training
Source: BMC Med Educ. 2026 Sep 19;26:1531. doi: 10.1186/s12909-026-10403-7 (PMC13613609; doi:10.1186/s12909-026-10403-7)
Supplement: Supplementary file 3 [file 12909_2026_10403_MOESM3_ESM.docx]

**Additional file 3:** Table 2. Selected exemplary quotations

Table 2. Selected exemplary quotations

| Main code | Sub Code | Quotation |
| --- | --- | --- |
| Drama Seminar vs. Online Tool | Evaluation of the Drama Seminar | *“(…) that one is allowed and must define their own boundaries. (…) I found that very, very valuable.” (P5)* |
|  |  | *“I think it’s genuinely the combination – the views of those who have already dealt with the subject in depth (…) but also the way students presented it, which sometimes felt more  relevant because they are closer to our own perspective.” (P1)* |
|  |  | *“I was glad that I wasn’t sitting at the front. Because I often feel overwhelmed in conversation situations in general and then in front of the whole group (…) knowing that it will be a difficult conversation situation even more so.” (P4)* |
|  |  | *“(…) I think that especially if you want to learn how to deal with a situation like this, it’s worth a lot more, simply because you’ve had the conversation yourself.” (P14)* |
|  |  | *“I think it would generally make sense to have some kind of guidelines not only for assisted suicide, but also for other, let’s say extreme situations, especially in communication (…).” (P15)* |
|  |  | *“(…) for some people it would be easier (…) to learn things if you really had a one-to-one contact.” (P13)* |
|  | Evaluation of the Online Tool | *“I can do it whenever I want (...) I find that incredibly pleasant.” (P6)* |
|  |  | *“(…) to be at home in a familiar environment (…) and not with strangers.” (P15)* |
|  |  | *“I think for someone who’s had no exposure to this at all, those preset answer options are incredibly valuable.” (P4)* |
|  |  | *“(…) I just didn’t retain much from it. I mean, it still feels like just another online tool, like any other one (…).” (P7)* |
|  |  | *“(…) because the answer options were fixed, it kind of felt more like a quiz (…).” (P9)* |
|  |  | *“Well, I think the closest thing would probably be a Zoom call.” (P10)* |
|  | Comparison of Both Formats of  Teaching | *“(…) because it’s only in the practical seminar that you can really express and learn empathy, which for me is absolutely central in the context of such a conversation.” (P10)* |
|  |  | *“(…) I think the fear of the real conversation wouldn’t be so strong, because you’d simply feel prepared.” (P4)* |
| Legal Framework | The Role of Legal Uncertainty | *“I find it somewhat difficult that it’s such a grey area, because having a clearly defined legal situation would make things easier. But banning it completely, I don’t think that’s good either. So, in that case, I prefer the grey area. But an official legalisation (…) would probably make it easier.” (P1)* |
|  | Proposals for Enhancing the Assisted Suicide Act | *“(…) I think this is a topic where we should really strive to do as much justice as possible to each individual person. (…) If you standardise things with fixed procedures and decision-making algorithms some people will inevitably fall through the cracks.” (P13)* |
|  |  | *“What I think is still missing are regulations, more specific regulations for, let’s say, people for whom assisted suicide doesn’t work in the usual way, because they’re, for example, completely paralysed or something like that. And I think there should be some kind of regulation that doesn’t just say it’s not allowed.” (P9)* |
|  |  | *“(…) that somehow you feel afraid of the responsibility, and if there were some kind of legal regulations for it, I think I would find that somehow reassuring.” (P15)* |
| Ethical Considerations in Assisted Suicide | Ethical Self-Determination and Values | *“I think, at least unconsciously definitely does have an impact. But on the other hand, I think I would even try to block it out for myself. Because if a 25-year-old is feeling really badly, then (…) of course they have the same desire to die as a 90-year-old or so. So, I don’t think it should matter, even if it might do.” (P14)* |
|  |  | *“(…) I’d probably draw my personal boundary somewhere between 60 and 70 years. By then, people have lived a good amount of time. That would be my emotional boundary, too, anything under 60, I find pretty intense.” (P6)* |
|  |  | *“(…) I must have the feeling that the person has given it a lot of thought (…). That it’s not a spontaneous whim and that they don’t seem desperate or under pressure but stable. (…) They have already taken care of things and somehow realised what it means.” (P5)* |
|  |  | *“I would say I support this idea promoting health (…) but I don’t fully stand behind it. I mean, sure. But in the end, (…) well, the wish also comes from a desire for what might be called freedom from pain. Or maybe for pain relief, or for dignity which itself can be seen as a form of health.” (P13)* |
|  | Balancing Responsibility and Emotional Demands | *“(…) that’s also a problem I have with physician-assisted death. That person can basically hand over the responsibility and say: “Go ahead and do it”. And in a way, that makes it easier, especially if someone isn’t really ready to take responsibility for themselves or for their own death.” (P5)* |
|  |  | *“Psychiatric diagnoses are too risky for me. I would have a problem with that. (…) Because something like severe depression or schizophrenia, I think, is the worst thing you can have. But I think I would personally be really scared to do something in a case where the patient can’t give informed consent. And how can you clearly define that? The patient must be in control of their mind, in a way.” (P4*) |
|  |  | *“I think you simply have to protect yourself.” (P4)* |
|  | (Free) Decision-Making | *“(…) the reality is that resources are limited, and spots in nursing homes and hospitals are scarce. And of course, in an ideal world, you’d hope that wouldn’t affect the patient. (…) But we’re not free from pressure either. That too remains an idealized assumption.” (P5)* |
| Teaching on Communication in the Context of Assisted Suicide |  | *“(…) considering we will be working in a profession that is extremely psychologically demanding, there is actually no course that teaches us how to cope with that (…).” (P13)* |
|  |  | *“The average student doesn’t really have any contact with patients yet, except maybe in examination courses. That’s why I don’t think it’s necessary to be exposed to palliative care at this stage.” (P8)* |
|  |  | *“I do think it is extremely valuable, within this context, to have such a conversation with a simulated patient, because it is (…) something entirely different from simply sitting in a lecture, possibly glancing at one’s phone from time to time and passively taking in the content. In a seminar that includes a simulation, one is, I believe, much more engaged, and consequently retains significantly more.” (P2)* |
|  |  | *“(…) I do think more teaching would be beneficial. I mean, the palliative care block is really very short, and especially regarding legal matters or even just an initial orientation or introduction – I think the broader this is, the better.” (P15)* |
|  |  | *“I think it would be useful, let’s say, to perhaps discuss these issues within the framework of an interdisciplinary subject or even separate course, generally speaking, perhaps as an additional ethics module.” (P9)* |
