# Supplementary material for: Finding the right words: preparing medical students for communication on the wish for hastened death - medical students’ perceptions of drama-based and online communication training
Source: BMC Med Educ. 2026 Sep 19;26:1531. doi: 10.1186/s12909-026-10403-7 (PMC13613609; doi:10.1186/s12909-026-10403-7)
Supplement: Supplementary file 4 [file 12909_2026_10403_MOESM4_ESM.docx]

**Additional file 4:** Semi-Structured Interview Guide: Finding the Right Words: Students’ Perspectives on Communication about Wishes for Hastened Death 

**Semi-Structured Interview Guide: Preparing Medical Students for Communication on the Wish for Hastened Death**

**1. Section: General Information**
1.1 Self- generated pseudonym code
1.2 How old are you?
1.3 Which semester are you currently studying in?
1.4 Why did you choose this elective?

**2. Section: Assisted Suicide**
2.1. What do you associate with the topic of assisted suicide?
- Thoughts, experiences, ideas, examples

**3. Section: Personal Experiences and Legal Framework**
3.1 Describe any personal experiences or experiences of others related to assisted suicide.
- Have you yourself been confronted with assisted suicide or a wish for hastened death?
- Where do you think you might encounter the topic of assisted suicide in your future professional life?
- What conditions would need to be met, and where would the boundary be drawn, in order to address patients’ wishes for assisted suicide?
- How do you perceive the current legal situation regarding assisted suicide in Germany?
- Would you wish for further legal regulation? If so, in what way?

**4. Section: Role of the Physician in the Context of Assisted Suicide**
4.1 How do you understand the role of the physician in the context of a patient’s request for assisted suicide?
- What aspects do you consider to be part of this role, and which do you not?
- Do you see assisted suicide as part of a physician’s professional responsibilities, and what kind of role, if any, do physicians have in this context?
 **5. Section: Perceived Effects of the Educational Interventions on Knowledge and Confidence**
5.1 How prepared do you feel to deal with patients’ wishes for death?
- What feelings do you associate with such requests?
- How do you evaluate your university’s preparation in this regard? Where do you see room for improvement?
- How confident do you feel, after the course, in informing patients about palliative care and options related to assisted suicide?
- Did participation in this course influence your views on the topic?
- What would you wish for in order to feel better prepared?

**6. Section: Perceptions and Evaluation of the Teaching Methods**
6.1 When you think about the drama-based method, what comes to mind?
- What do you remember most vividly?
- What did you like? What did you not like as much?
- What suggestions for improvement do you have?

6.2 When you think about the online tool, what comes to mind?
- What do you remember most vividly?
- What did you like? What did you not like as much?
- What suggestions for improvement do you have?

6.3 When you compare the online tool and the drama-based method, which one did you prefer?
- Can you describe what most strongly influenced or shaped your opinion?
- How did your role (observer vs. active participant) shape your perception of the drama-based scenario? How do you think your experience would have differed in the other role?

**7. Section: Reflection**
7.1 Is there anything else you would like to add?
- Suggestions for the course
- Positive or negative feedback
